# Implementation of independent nurse prescribing in UK mental health settings: focus on attention-deficit/hyperactivity disorder

**DOI:** 10.1007/s12402-014-0138-x

**Published:** 2014-04-18

**Authors:** Lisa Mangle, Paula Phillips, Mark Pitts, Cathy Laver-Bradbury

**Affiliations:** 1 Shire, Unity Place, Hampshire International Business Park, Basingstoke, RG24 8EP UK; 2 Forensic CAMHS, Wakefield, West Yorkshire UK; 3 Adult ADHD Service, Maudsley Hospital, London, UK; 4 CAMHS Solent NHS Trust, Southampton, UK; 5 Southampton University, Southampton, UK

**Keywords:** Attention-deficit/hyperactivity disorder, Non-medical prescribing, Controlled drugs, Nurse-led services, Nurse independent prescribing

## Abstract

Legislative changes that came into effect in the UK in April 2012 gave nurse independent prescribers (NIPs) the power to prescribe schedule 2–5 controlled drugs. Therefore, suitably qualified UK nurses can now independently prescribe any drug for any medical condition within their clinical competence. The potential benefits of independent nurse prescribing include improved access to medications and more efficient use of skills within the National Health Service workforce. This review explores the published literature (to July 2013) to investigate whether the predicted benefits of NIPs in mental health settings can be supported by empirical evidence, with a specific focus on nurse-led management of patients with attention-deficit/hyperactivity disorder (ADHD). The most common pharmacological treatments for ADHD are controlled drugs. Therefore, the 2012 legislative changes allow nurse-led ADHD services to offer holistic packages of care for patients. Evidence suggests that independent prescribing by UK nurses is safe, clinically appropriate and associated with high levels of patient satisfaction. The quality of the nurse–patient relationship and nurses’ ability to provide flexible follow-up services suggests that nurse-led ADHD services are well positioned to enhance the outcomes for patients and their parents/carers. However, the empirical evidence available to support the value of NIPs in mental health settings is limited. There is a need for additional high-quality data to verify scientifically the value of nurse-delivered ADHD care. This evidence will be invaluable in supporting the growth of nurse-led ADHD services and for those who support greater remuneration for the expanded role of NIPs.

## Introduction

In April 2012, UK legislative changes gave nurse independent prescribers (NIPs) the power, for the first time, to prescribe schedule 2–5 controlled drugs (Table 1). Nurse prescribing from a limited formulary was first introduced in the UK in the 1990s, and in 2001, this was extended to include a larger range of medicines. In 2002, supplementary prescribing was introduced; this is a voluntary partnership between an independent prescriber (doctor or dentist) and a supplementary prescriber (e.g. a nurse) and, with the patient’s agreement, allows the supplementary prescriber to prescribe medicines within an agreed clinical management plan. Supplementary prescribing was extended to include controlled drugs in 2005. The introduction in 2006 of independent prescribing enabled nurses to prescribe any medication from the British National Formulary, with the exception of controlled drugs. The 2012 legislative changes mean that suitably qualified nurses (Table 2) can now independently prescribe any drug, including schedule 2–5 controlled drugs, for any medical condition within their clinical competence. As a result of this series of legislative changes, nurses in the UK have the most extensive prescribing rights of nurses worldwide. Currently, there are approximately 20,000 NIPs registered with the Nursing and Midwifery Council in the UK (Stewart et al. 2013). Many believe that nurse prescribing in the UK improves access to medications for patients by increasing the availability of healthcare providers who can prescribe, and represents a more efficient use of skills through utilising the existing expertise within the nursing workforce (Latter et al. 2011).

**Table 1:** Changes to the misuse of drugs regulations 2001 (April 2012)

Changes relating to independent prescribing of controlled drugs in the UK came into force on 23 April 2012 [Misuse of Drugs (Amendment No. 2) (England, Wales and Scotland) Regulations 2012 (Statutory Instrument 2012/973)]
Previous restrictions were removed, allowing independent nurse prescribers to:
Prescribe any controlled drug listed in schedules 2–5 for any medical condition within their competence, with the exception of diamorphine, cocaine and dipipanone for the treatment of addiction
Requisition schedule 2–5 controlled drugs and to possess, supply, offer to supply and administer them
Mix any drugs listed in schedules 2–5 before administration
These rights also apply to pharmacist independent prescribers
Persons acting in accordance with the directions of a nurse or pharmacist independent prescriber are authorised to administer schedule 2–5 drugs

**Table 2:** Nurse independent prescribing: UK training requirements

Nurses must complete a Nurse and Midwifery Council-accredited prescribing course through an Approved Education Institution (AEI; a UK university) that:
Includes a minimum of 26 days of teaching
For distance-learning programmes, there must be a minimum of eight face-to-face taught days
Includes 12 days of supervised learning in practice
Is provided at a minimum of first degree level (academic level 3)
AEIs may offer the course at Masters level
Is completed within one academic year
To be eligible to undertake an accredited nurse prescribing course, nurses must:
Be a registered first-level nurse, midwife and/or specialist community public health nurse
Have at least 3 years’ experience as a practising nurse, midwife or specialist community public health nurse
The year immediately preceding the programme must have been in the clinical field in which they intend to prescribe
Be deemed competent by their employer to undertake the programme
The employer is responsible for confirming that each nurse is competent in history-taking, clinical assessment and diagnosing, and possesses appropriate numeracy skills
The employer must provide written confirmation of their support for each nurse to undertake the course
Provide evidence via the Accreditation of Prior and Experiential Learning process of their ability to study at degree level
Obtain written confirmation from the course lead about their acceptance on the course
Obtain written confirmation from a designated medical practitioner who meets eligibility criteria for medical supervision of nurse prescribers and who has agreed to provide supervised practice

In this review, we explore the impact of the extended prescribing rights of UK NIPs on nurse-led services for patients with attention-deficit/hyperactivity disorder (ADHD) and examine the potential benefits of expanding the delivery of nurse-led ADHD services in the UK. In 2005, a Cochrane review evaluated the impact of substituting nurses for doctors in primary care, concluding that appropriately trained nurses can provide care of equally high quality to that provided by primary care doctors and can achieve equally good health outcomes for patients (Laurant et al. 2005). However, the authors urged caution in interpreting their conclusions because data from high-quality studies were very limited. Furthermore, this earlier review included studies published from 1966 to 2002 and was not limited to UK studies; hence, it is unclear how applicable these findings are to the current UK nursing environment. Here, we examine the published literature (to July 2013) to investigate whether the predicted benefits of nurse independent prescribing in mental health settings can be supported by empirical evidence, with a specific focus on the management of patients with ADHD. Approaches to the further development of nurse-led ADHD services in the UK are also discussed.

### Introduction to ADHD

ADHD is one of the most common neurodevelopmental disorders in children, affecting both boys and girls, and continuing into adulthood in many patients (Polanczyk et al. 2007; Willcutt 2012). The disorder is characterised by persistent symptoms of hyperactivity/impulsivity and/or inattention and, in the long term, is associated with significant impairments in multiple aspects of the patient’s life, including academic performance, employment status, interpersonal relationships, financial planning and legal issues (Mattingly 2011; Coghill et al. 2008). Managing and supporting patients with ADHD requires a multimodal approach and involves interactions with multiple groups, for example, patients’ families, educational establishments, employers and the criminal justice system (National Institute for Health and Clinical Excellence 2009; Nutt et al. 2007). Optimal management of ADHD aims not only to minimise the symptoms of ADHD but also to reduce the impairments associated with the disorder. When pharmacological treatment for ADHD is indicated, the most commonly used agents, both in the UK and worldwide, are stimulant medications, which are schedule 2 controlled drugs (Garfield et al. 2012; Knopf et al. 2012; McCarthy et al. 2012). Therefore, UK nurses can now independently provide a complete and holistic package of care for individuals with ADHD as a direct result of the 2012 legislative changes.

## Do UK nurses have the skills required to diagnose ADHD?

### General principles of diagnosing ADHD

Like many mental health conditions, ADHD must be diagnosed clinically; there are no definitive and objective tests (e.g. genetic or neuropsychological tests). Diagnosis can also be complicated by the fact that patients with ADHD often have co-existing psychiatric disorders. However, high levels of diagnostic certainty can be achieved through use of diagnostic rating scales in combination with a developmental history, observation, and the assessment of family and other risk factors (Nutt et al. 2007). According to the UK’s National Institute for Health and Care Excellence (NICE) guidelines, for a diagnosis of ADHD, symptoms of hyperactivity/impulsivity and/or inattention should meet the diagnostic criteria in the *Diagnostic and Statistical Manual of Mental Disorders, Fourth Edition* (DSM-IV) or the *International Statistical Classification of Diseases and Related Health Problems, 10th Revision* (ICD-10) (hyperkinetic disorder), be associated with at least moderate psychological, social and/or educational or occupational impairment and occur in two or more important settings including social, familial, educational and/or occupational settings (National Institute for Health and Clinical Excellence 2009). NICE guidelines have yet to be updated to reflect the recently published *Diagnostic and Statistical Manual of Mental Disorders, Fifth Edition* (DSM-5), but it is noteworthy that DSM-5 differs from its predecessor in requiring several inattentive or hyperactive symptoms to be present before 12 years of age, compared with the presence of symptoms that cause impairment prior to the age of 7 years in DSM-IV (American Psychiatric Association 2013). Based on the high degree of clinical judgement required to make a diagnosis, expertise in the field of ADHD is needed in order for both doctors and nurses to make accurate diagnoses of the condition.

### Independent diagnosis by UK nurses: lessons from other therapy areas

Nurses diagnose various medical conditions in clinical practice. In a UK study, 73.8 % of NIPs surveyed (*n* = 862), working across a range of therapy areas, reported that they made the diagnosis on most occasions in their prescribing practice (Latter et al. 2011). Despite this, the number of studies assessing the accuracy of diagnosing by UK nurses in any disease area is currently limited. The ability of nurses to diagnose headache disorder to the same standard as doctors was established in a small UK study involving one neurology ward sister, trained in the differential diagnosis of headache disorder, and three consultant neurologists (Clarke et al. 2005). Following examination of 239 patients and 13 role-players, the doctor’s primary diagnosis was reached by the nurse in 90 % of cases of headache and migraine, and 61 % of other diagnoses (Clarke et al. 2005). Of the 30 cases in which the nurse did not reach the same diagnosis as the doctor, 22 were referred for further evaluation. Examination of patients’ records at least 6 months after participation indicated that no sinister headache pathologies were missed, suggestive of high quality diagnosing by the nurse and the consultants (Clarke et al. 2005).

The quality of nurse diagnosing was also examined in a retrospective case note study, which examined the records of 404 UK patients diagnosed with dementia (Page et al. 2008). The study compared the initial diagnoses of two nurses with subsequent formal diagnoses at a multidisciplinary memory clinic, revealing that the nurses accurately detected dementia in 94 % of cases (Page et al. 2008). However, interpretation of both of these studies is limited owing to the very small numbers of nurses involved.

The ability of nurses to diagnose medical conditions accurately is further supported by two larger studies (Latter et al. 2011, 2012; Naughton et al. 2012). The first reviewed 100 audio-recordings of consultations by NIPs (*n* = 52) and pharmacist independent prescribers (PIPs; *n* = 48) using the Medication Appropriateness Index (MAI; Latter et al. 2011, 2012). In 87.5 % of cases reviewed, an ‘appropriate’ rating was awarded for the MAI item ‘Is there an indication for the medication?’ suggestive of appropriate diagnosing practice (Latter et al. 2011, 2012). The largest number of comments provided by the MAI raters was in praise of the prescribing episode (88/272 comments; Latter et al. 2011, 2012). However, the second most frequent group of comments related to the potential for improvements in independent prescribers’ history-taking, assessment and diagnosis skills (52 comments; Latter et al. 2011, 2012). In agreement with this study, an MAI study of nurse prescribers in Ireland (including 142 patient records) found that the prescribed medication was appropriately indicated in 95 % of cases (Naughton et al. 2012). Therefore, evidence is available to support the idea that UK nurses can diagnose medical conditions accurately, but further research is required to endorse this claim.

### Independent diagnosing of ADHD by nurses

There are currently no studies that have examined the ability of UK nurses to diagnose ADHD accurately; however, two studies were identified that examined the ability of non-UK-based nurses to diagnose ADHD. First, a US-based study concluded that advanced practice registered nurses followed American Academy of Pediatrics diagnostic guidelines for ADHD in children more closely than did other healthcare providers (paediatricians and family physicians; Vlam 2006). Of the nurses surveyed, 52 % were confident in making an ADHD diagnosis (Vlam 2006); however, interpretation is limited as the study did not report the proportion of clinicians who were confident in diagnosing ADHD. Low levels of confidence in diagnosing ADHD in children and adults were found in a second study of US nurse practitioners (Knutson and O’Malley 2010). However, conclusions from this study are also severely limited because 74.3 % of nurses included in the study indicated that they had never diagnosed ADHD in children, and 66.5 % had never diagnosed the disorder in adults. Clearly, empirical evidence to establish the accuracy of ADHD diagnosing by UK nurses is lacking, and if the provision of nurse-led ADHD services were to be expanded, this is an area in which further research is needed.

## Is independent prescribing by UK nurses safe and appropriate?

### Safe and clinically appropriate prescribing

Several studies in the UK and Ireland have found that non-medical prescribing (which includes prescribing by nurses and pharmacists) is safe and clinically appropriate (Black 2013; Latter et al. 2011, 2012; Naughton et al. 2012). Latter et al. (2012) reviewed 100 audio-recordings of consultations using the MAI, concluding that NIPs (*n* = 52) and PIPs (*n* = 48) were making clinically appropriate prescribing decisions (Latter et al. 2011, 2012). High levels of ‘appropriate’ rating were awarded for most MAI items (range 79–93.5 % for 9/10 items), with the lowest score recorded for the item ‘Is this drug the least expensive alternative to others of equal utility?’ (69.5 %) (Latter et al. 2011, 2012). This study states that their findings are broadly consistent with those from MAI studies investigating doctors’ prescribing decisions, but urges caution owing to the lack of directly comparable studies (Latter et al. 2012). The MAI was also used to evaluate clinical appropriateness and safety of nurse and midwife prescribing in Ireland, concluding that 69–80 % of prescribing episodes were deemed appropriate across all eight MAI items assessed (Naughton et al. 2012).

A retrospective review of 764 NIPs’ case notes in accident and emergency and sexual health concluded that prescribing practice was safe in 99.4 % of cases (*n* = 566) (Black 2013). However, improved documentation was required in eight cases, and in two of these, the prescribing episode was deemed unsafe owing to the lack of documented past medical history (Black 2013). As with the other studies described here, comparisons with medical colleagues were not included in this study, making the findings difficult to interpret. No studies were identified that specifically examined the safety or clinical appropriateness of independent nurse prescribing for ADHD, in the UK or elsewhere. This is not surprising as UK nurses have been able to prescribe stimulants independently, without the need for a clinical management plan, only since April 2012. Therefore, data regarding the safety or clinical appropriateness of independent nurse prescribing for ADHD in the UK have yet to become available. The generation of these data will be highly valuable in guiding the future expansion of nurse-led ADHD services.

### Prescriber confidence

Studies indicate that NIPs have high levels of confidence in their prescribing abilities (Gumber et al. 2012; Latter et al. 2011). Only 6 % of 862 UK NIPs surveyed (working across a range of therapy areas including mental health) had concerns that they were prescribing outside their competency, compared with 9.8 % of 143 UK PIPs (Latter et al. 2011). In addition, only 4.0 % of NIPs, compared with 10.5 % of PIPs, agreed with the statement ‘I do not have the pharmacological knowledge to be a safe independent prescriber’ (Latter et al. 2011). It is notable, however, that 58 % of NIPs surveyed, compared with 28.2 % of PIPs, expressed concerns about prescribing for patients with comorbidities (Latter et al. 2011). This point is particularly pertinent to ADHD treatment as the disorder is frequently associated with psychiatric comorbidities.

In a second UK study, all 20 mental health non-medical prescribers surveyed (which included 18 nurses) believed that they worked within their competency, and most agreed that they had adequate knowledge of side effects, dosage, drug interactions, withdrawal effects and physical health monitoring (Gumber et al. 2012).

The NIP qualification requires a minimum of 12 days of supervised learning in practice by a designated medical practitioner. In a survey of 840 NIPs, this supervised practice was considered a highly positive experience and 55.0 % of NIPs reported receiving more than the 12 days required of supervised practice (Latter et al. 2011). The use of clinical supervision is likely to enhance the confidence of NIPs and also provides quality assurance.

### Attitudes among medical colleagues

Studies investigating the opinions of UK doctors towards independent nurse prescribing in mental health settings have yielded mixed results. In semi-structured interviews, two psychiatrists with experience of working with NIPs expressed positive views on the competency of NIPs and the benefits they can bring to patient care (Earle et al. 2011b). Both psychiatrists, however, stressed the importance of adequate supervision (Earle et al. 2011b). In a larger study involving 147 psychiatrists, just over half of consultants felt that mental health nurses should be able to prescribe independently, whereas 80 % of junior doctors felt that they should not (Rana et al. 2009). In contrast, significantly more junior doctors than consultants felt that nurses should be able to prescribe out of hours and at weekends (Rana et al. 2009). Only 20 % of consultants and none of the junior doctors surveyed felt that nurses should be able to prescribe controlled drugs (Rana et al. 2009). Notably, across the entire survey, senior psychiatrists had fewer concerns about the creation of new nursing roles than their junior colleagues, possibly reflecting higher levels of anxiety among junior doctors (Rana et al. 2009).

In apparent contrast to these findings, 87 % of NIPs believed that the doctors they worked with were supportive of nurse independent prescribing in a more recent UK study that was not restricted to mental health settings, and 54 % agreed that independent prescribing had increased the respect they received from doctors (Latter et al. 2011). It is possible that these discrepancies reflect rapidly changing viewpoints as the role of non-medical prescribers expands and doctors gain greater experience of working with NIPs, or they may reflect a particular reluctance towards non-medical prescribing in mental health settings.

## Treating and managing patients with ADHD in the UK

### ADHD treatments

In the UK, psychoeducational and behavioural interventions are recommended as first-line treatments for children and adolescents with mild or moderate impairment resulting from ADHD (National Institute for Health and Clinical Excellence 2009). These include parent-training/education programmes and group treatment programmes for the patient (cognitive behavioural therapy and/or social skills training). Pharmacological treatment is generally reserved for children and adolescents with more severe symptoms. Stimulants (methylphenidate- and amphetamine-based formulations) and the non-stimulant atomoxetine are approved medications for the treatment of ADHD in children and adolescents in the UK, with methylphenidate the most commonly prescribed medication (National Institute for Health and Clinical Excellence 2009). For adults in the UK, pharmacological treatment, and specifically methylphenidate, is recommended as first-line therapy (National Institute for Health and Clinical Excellence 2009). Stimulants are schedule 2 controlled drugs; hence, the recent legislative changes allow UK NIPs to prescribe any of the licensed ADHD medications independently. The evidence presented in the previous section suggests that nurses are equipped to expand their role to include independent prescribing of stimulants. As mentioned earlier, powers to prescribe stimulants were only recently granted to NIPs, and as such, there is as yet no empirical evidence available to either support or refute this suggestion. Establishing the safety and clinical appropriateness of NIP-controlled drug prescribing, including ADHD medications, is highly desirable.

### Managing patients with ADHD

Managing and supporting patients with ADHD involves interactions with multiple groups, including parents and other carers, teachers/lecturers and the criminal justice system. These interactions are time-consuming and require flexibility. For those patients receiving ADHD medication additional, regular monitoring is required, for example, measuring height, weight, blood pressure and pulse, assessing side effects, determining the efficacy of treatment, deciding whether dose adjustments or treatment switches would be beneficial, and establishing the need for continued treatment (National Institute for Health and Clinical Excellence 2009).

Support is also needed to enhance adherence to treatment regimens because, like other chronic health conditions, adherence to ADHD medication is low; a review of 11 studies in children and adults with ADHD reported mean rates of non-adherence to medication of 13–64 % (Adler and Nierenberg 2010). Adherence can be particularly problematic in adolescents and adults as parental supervision is reduced, with these individuals particularly vulnerable to dropping out of ADHD services during transitions from paediatric to adult care (Taylor et al. 2010). As a result, these patients require even greater levels of support from healthcare providers. Given the chronic nature of ADHD, non-adherence to treatment can be a major determinant of treatment outcome and can have huge health, social and economic implications.

### The role of nurse-led services in managing patients with ADHD in the UK

The available data, discussed below, indicate that nurses can deliver high-quality care for patients with ADHD and can enhance the experience of service users owing to the unique nurse–patient relationship. The particular aspects of nurse-led care that we predict are most likely to lead to improved patient satisfaction and outcomes are outlined in Table 3.

**Table 3:** Opportunities for nurse-led services to enhance ADHD care

Independent prescribing: timely access to treatment
Nurses can initiate new treatment or modify the dose/formulation of medication instantly without the need to refer to a clinician
Patient care can be improved by allowing rapid balancing of symptom control and side effects
Continuity of care
Nurses can deliver a complete, multimodal care package
Nurses can regularly interact with individuals involved in patient care (e.g. parents, schools and the criminal justice system) in clinical and non-clinical settings
Nurses can get to know patients and their families over an extended period of time, facilitating individualised treatment strategies
Longer and more frequent appointments
Nurses can provide increased consultation time, facilitating greater patient understanding of treatment regimens; this is linked to improved adherence to treatment
Named nurse contact details
Patients, parents, schools and other relevant parties can obtain rapid guidance on the treatment of individual patients
Patient and parent support
Nurses can run support groups and drop-in clinics for patients and/or parents
Nurse–patient relationship
Patients and parents may feel less intimidated by nurses than by clinicians, thus facilitating open and honest discussion of their symptoms/behaviours and treatment adherence

#### Increasing client satisfaction

Several studies indicate that there are high levels of satisfaction with nurse prescribing in UK mental health settings among clients (which include patients and carers; Earle et al. 2011a; Laver-Bradbury and Harris 2009; Ross et al. 2013). Five out of six patients interviewed did not feel that there were any disadvantages or concerns regarding nurse prescribing in a survey of patients from one UK mental health National Health Service (NHS) trust (Earle et al. 2011a). Patients particularly valued the convenience of home visits and believed that nurses provided longer and more frequent appointments than doctors. Patients also reported feeling less intimidated speaking to a nurse than a doctor, highlighting the importance of the unique nature of the nurse–patient relationship. Two patients recognised, however, that some individuals may prefer to see a doctor and believed that doctors had greater knowledge than nurses (Earle et al. 2011a). Similarly, high levels of satisfaction with nurse prescribing were found among nine clients from another UK mental health NHS trust, with many clients believing that nurse prescribing had strengthened the nurse–patient relationship and had improved access to care (Ross et al. 2013).

Parents (*n* = 12) of UK-based patients with ADHD were found to be at least as satisfied with the care received from ADHD advanced nurse practitioners as with that previously provided by medical staff (Laver-Bradbury and Harris 2009). This study also found that parental views on support groups (*n* = 28) were almost entirely positive. Some of the parents completed the General Health Questionnaire (GHQ; *n* = 15) and the Parenting Sense of Competency Questionnaire (PSOC; *n* = 13) before and after attending support groups. Using the GHQ, 12 parents had an initial score greater than 12 (indicating health problems), whereas, 4 weeks after starting to attend the support group, only six parents had a score greater than 12 (Laver-Bradbury and Harris 2009). In addition, there were statistically significant improvements in mean PSOC ‘total’ and ‘satisfaction’ scores 4 weeks after starting to attend the support group, indicating improved levels of parenting competency and satisfaction (Laver-Bradbury and Harris 2009). The same study collected parental feedback on nurse-led drop-in clinics, revealing that this service was highly valued by parents who particularly appreciated the access to professional advice and support between clinical appointments, and the flexibility to attend at a time that was convenient for them (Laver-Bradbury and Harris 2009).

Parental views were also investigated in a Canadian continuity of care study that sought opinions of parents of children with complex chronic health conditions, including children with ADHD (Miller et al. 2009). This study indicated that parents highly valued care in which knowledge of the child was developed through relationships with a consistent set of service providers, both inside and outside medical settings (Miller et al. 2009).

Nurses are in a position to offer flexible services to patients with ADHD and their parents, and can also interact with non-medical service providers involved in provision of patient care (e.g. teachers). The legislative changes permitting the prescribing of controlled drugs by NIPs provide the opportunity for nurse-led ADHD services to offer a complete package of care to individuals with ADHD, facilitating enhanced engagement with patients and carers and improved continuity of care, which would be expected to lead to increased levels of client satisfaction.

#### Promoting adherence to treatment

As with other chronic health conditions, improving adherence to treatment is key to maintaining effective control of symptoms and problem behaviours associated with ADHD. Adherence to ADHD medications is consistently higher among patients involved in clinical trials than among those receiving treatment in the community (Chacko et al. 2010). These observations provide indirect evidence that longer and more frequent follow-up appointments may lead to increased adherence among patients with ADHD. Interventions that have been identified to enhance adherence to long-term treatment in general include combinations of the following: more convenient care; information; counselling; reminders; self-monitoring; reinforcement; family therapy; psychological therapy; mailed communications; crisis intervention; manual telephone follow-up; and other forms of extra supervision or attention (Haynes et al. 2008).

No studies were identified that assessed the impact of nurse-led care on adherence specifically to ADHD medication. However, several studies have demonstrated that nurse-led interventions can improve adherence in other therapy areas. A systematic review of articles published from 2006 to 2011 identified 10 randomised controlled trials on nurse-led interventions aimed at improving chronic medication adherence in adults (Van Camp et al. 2013). Included studies were conducted in the USA (*n* = 5), Europe (*n* = 3), Asia (*n* = 1) and Africa (*n* = 1), with seven studies focused on patients who were HIV-positive and the remaining three assessing patients with depression, arthritis and hypertension (Van Camp et al. 2013). Most interventions were multifactorial, combining counselling with education, social support and practical help (e.g. text message reminders). Nurse-led interventions enhanced short-term adherence in nine studies (results were statistically significant in four studies), and the intervention effect was sustained in the long term (3–12 months post-intervention) in eight studies (results were statistically significant in four studies; Van Camp et al. 2013).

In a randomised UK-based study assessing adherence to inhaled combination therapy in adult patients referred to a Regional Difficult Asthma Service, a nurse-delivered intervention strategy led to significantly increased adherence at 12 months relative to the control group (Gamble et al. 2011). There was also a non-significant trend towards fewer hospital admissions in the intervention group at 12 months. In addition, a randomised UK-based trial in adult patients with type 2 diabetes found that a nurse-delivered consultation-based intervention increased objectively measured medication adherence (Farmer et al. 2012).

### How effective are UK-based nurse-led care models for ADHD?

In the UK, nurse-led services are being used effectively to manage a variety of health conditions (e.g. diabetes, pain management and smoking cessation). However, the data available to assess the quality of nurse-delivered management of patients with ADHD are, so far, limited. The quality of nurse-led and doctor-led management was compared in patients with ADHD who had been previously diagnosed and titrated to a stable medication dose by a doctor (Foreman and Morton 2011). The study, which included two doctors and one ADHD nurse, indicated that all three individuals provided very similar standards of care when assessing symptomatic change, side-effect burden and service user satisfaction (Foreman and Morton 2011). Care delivered by nurses was found to be as good as that delivered by doctors even in more complex cases of ADHD (Foreman and Morton 2011). However, interpretation of these data is limited owing to the low numbers of healthcare providers included in the study.

Contact with UK schools was enhanced when they were provided with a specific named nurse to contact regarding the management of a child with ADHD, and schools reported that the provision of nurse-based intervention had been beneficial to the children (Laver-Bradbury and Harris 2009). The same study also indicated that nurse-led interventions led to improvements in patient outcomes (Laver-Bradbury and Harris 2009). Parents completed the Strengths and Difficulties Questionnaire (SDQ; *n* = 13) before and after attending nurse-led support groups (over a 4-week period; Laver-Bradbury and Harris 2009). The results of the SDQ, which assesses changes in parents’ reports on their child’s strengths and difficulties, revealed statistically significant improvements after attending the support groups in the ‘hyperactivity’ and ‘peer problems’ domains (though not in ‘emotional symptoms’, ‘conduct’ or ‘prosocial behaviour’ domains), indicating that the flexible working practices that can be employed by nurses not only may enhance parent satisfaction (described previously), but also may lead to some improvements in patient outcomes (Laver-Bradbury and Harris 2009).

Therefore, initial evidence obtained in ADHD and other therapy areas indicates that nurse-led services are very well placed to provide high-quality, complete care packages for patients with ADHD, and it is hoped that further empirical evidence will be generated to confirm this prediction.

### Relevance of findings to non-UK healthcare settings

This review specifically focuses on the empirical evidence available to support the potential benefits of nurse-led ADHD services in the UK healthcare setting, where nurses currently have the most extensive prescribing rights of nurses worldwide. Outside of the UK, the ability of nurses to prescribe controlled ADHD medications varies. For example, it is not permitted at all in Germany, whereas in Australia controlled drug prescribing is permitted by a subset of nurses working in rural and isolated practice. Given the fact that widespread controlled drug prescribing by nurses is not generally permitted in countries other than the UK, nurse-led ADHD services outside of the UK would not currently be able to offer the holistic care packages possible for UK services. However, many aspects of these findings are likely to be relevant to nurses in other countries, and as nurse-led prescribing becomes increasingly embedded in the UK health system, healthcare providers elsewhere are likely to draw on the UK’s experiences in expanding the nursing role to better utilise the existing skills within their own nursing workforces.

## Cost-effectiveness of independent nurse prescribing

It has been suggested that independent prescribing by nurses allows existing skills within the nursing sector to be implemented and utilised more efficiently; this allows doctors’ time to be spent on more complex cases, thus providing economic benefits (Latter et al. 2011). However, the evidence to support these claims remains limited. In the 2005 Cochrane review, the authors agreed that doctor–nurse substitution had the potential to reduce healthcare costs (Laurant et al. 2005). However, they suggested that savings were likely to be highly dependent on the salary differential between doctors and nurses, and may be offset by lower productivity of nurses compared with doctors (Laurant et al. 2005).

More recently, a UK study performed a cost-minimisation analysis using an infection vignette (Latter et al. 2011). This study concluded that a combined general practitioner (GP) and NIP service for infection was less expensive than a traditional GP-only service at practice and primary care trust levels (training costs not considered; Latter et al. 2011). Cost benefits were also reported when nurse prescribers, rather than doctors, reviewed and monitored medication for patients with ADHD in the UK, even though patients were seen more often after they were transferred to nurse care (Laver-Bradbury and Harris 2009). Although cost-effectiveness was not assessed directly, another UK study concluded that the management of patients with ADHD by nurses was of a similar standard to that provided by doctors and suggests that nurse-led care would substantially reduce costs with no loss of quality (Foreman and Morton 2011). Therefore, based on available data, it appears likely that nurse-led ADHD services could provide cost benefits compared with clinician-delivered care, but this prediction has yet to be fully supported by empirical evidence. If nurse-led services resulted in improved outcomes for patients with ADHD (in terms of impairments across their lifespan), there would be far-reaching cost benefits to society as well as reduced symptoms and improved quality of life for the individual.

Care must be taken, however, that a desire for cost-effectiveness does not overshadow the requirement for a safe and effective ADHD service. It must be ensured that all staff, whether working in a nurse-led service or not, have adequate training in the multifaceted process of diagnosing and treating ADHD. In addition, it would seem wise that nurse-led ADHD services should remain affiliated with medical colleagues to aid in the care of patients with complex needs and to assist in the management of adverse events that may occur as a result of pharmacological treatment for ADHD.

## Barriers to implementation of independent nurse prescribing in mental health settings

In the UK, barriers to implementation of nurse prescribing in mental health settings include lack of support, role conflict with other healthcare professionals, concerns about the effect on the relationship with patients, concerns about the level of training and lack of recognition for additional responsibilities (via job status and remuneration; Earle et al. 2011a; Gumber et al. 2012; Jones et al. 2010; Ross and Kettles 2012).

### Lack of support

Despite legislative changes permitting independent prescribing by nurses, some NHS Trusts have not implemented this widely. A survey investigating barriers to prescribing by UK mental health NIPs (*n* = 33) found that only half of respondents felt supported in their role (Ross and Kettles 2012). Lack of medical supervision, clear prescribing policy and clinical governance were identified as further barriers (Ross and Kettles 2012). NIPs were also frustrated by the influence of nurse management on their prescribing role and suggested that nurse managers did not have sufficient knowledge of prescribing to warrant such influence (Ross and Kettles 2012). It should be noted that, despite being appropriately trained, 58 % of NIPs included in this study had never prescribed independently, perhaps resulting in particularly negative viewpoints.

### Insufficient training

Some studies have raised concerns about whether UK nurses are equipped with the required knowledge of pharmacology (Jones et al. 2010), numerical skills, drug calculation abilities (McMullan 2010) and competence in pharmacovigilance (Stewart et al. 2013) to fulfil the additional responsibilities associated with independent prescribing. Despite the fact that 76 % of mental health NIPs surveyed in the study by Ross and Kettles (2012) felt confident to prescribe, 55 % did not feel that the prescribing course had prepared them to do so (Ross and Kettles 2012). This view was shared in another survey of non-medical prescribers (*n* = 20) working in UK mental health settings, which found that 60 % agreed that there were shortfalls in training and supervision (Gumber et al. 2012). However, this finding is difficult to interpret owing to the fact that 70 % of responders agreed that they personally had received adequate supervision, with 80 % agreeing that they themselves had received adequate training in prescribing (Gumber et al. 2012).

As a possible solution to the potential training shortfall, one study of UK mental health NIPs (*n* = 13) demonstrated that a 10-day psychopharmacology course, costing £300 per participant, led to statistically and clinically significant gains in knowledge (Jones et al. 2010). The authors proposed that a portion of the prescribing course should be dedicated to specific training according to the disease area in which the trainee NIPs intend to practise (Jones et al. 2010). A study of ADHD nursing in the UK also identified a need for expansion of the nurse prescribing course to include children and young people’s mental health (Laver-Bradbury and Harris 2009). Jones et al. (2010) suggest that a greater understanding of psychopharmacology may increase the confidence of mental health nurses who hold the prescribing qualification, thus increasing the numbers who choose to use their prescribing qualification in practice (Jones et al. 2010). This opinion would appear to be supported by Ross and Kettles (2012), who found that nurses with higher qualifications were more confident to prescribe independently (Ross and Kettles 2012).

### Lack of recognition

Many NIPs believe that the additional levels of expertise and responsibilities associated with their prescribing role should be recognised in their job description and financially rewarded (Earle et al. 2011a; Jones et al. 2010; Ross and Kettles 2012). NIPs have also expressed the concern that the expansion of non-medical prescribing is politically motivated, suggesting that they were being paid cheaply to do the same job as doctors (Ross and Kettles 2012).

### Overcoming barriers to prescribing

Despite these identified barriers and reservations, there is evidence to indicate that NIPs who are actively prescribing have higher levels of job satisfaction than those who are not (Cousins and Donnell 2012; Ross and Kettles 2012). In a Department of Health evaluation, 54.2 % of NIPs indicated that the decision to become a prescriber had been driven by their own personal choice, with 38.5 % reporting that it was a joint decision with their employer (Latter et al. 2011). This may suggest that self-motivated individuals and those who are supported by appropriate management and service structures are able to overcome the perceived barriers to independent prescribing. Improving the professional support for newly qualified NIPs may allow greater numbers of trained NIPs to overcome these barriers to prescribing, reducing the waste of NHS resources associated with training nurses to prescribe who do not then feel able to do so.

## Conclusions

The symptoms and functional impairments associated with ADHD can negatively impact many aspects of patients’ lives and also present challenges for their parents, carers, teachers and employers. ADHD can be effectively managed, but this requires a multimodal treatment approach which can be time-consuming and labour intensive for healthcare providers. Legislative changes that came into effect in April 2012 permit UK nurses to prescribe schedule 2–5 controlled drugs, including stimulant medications for the treatment of ADHD, independently. This means that nurse-led services in the UK can now provide a full package of care for patients with ADHD.

We believe that UK ADHD nurses possess the required specialist skills and experience to deliver high-quality, holistic care for patients with ADHD. We also believe that nurse-led services have the potential to enhance the experience of patients with ADHD and their carers, and to deliver improved patient outcomes compared with standard care models. While multiple small-scale studies support the potential benefits of nurse independent prescribing, there are few large-scale studies and even fewer studies that directly compare nurse-delivered care with that of medical colleagues. Importantly for this review, none of the identified large-scale studies have specifically assessed nurse-delivered care for patients with ADHD, with evidence limited to a few small-scale studies. Latter et al. (2011) indicated that the expansion of nurse-led services to date has been largely driven by individual practitioners (Latter et al. 2011); we urge ADHD nurses in the UK also to drive the collection of high-quality study data, and the publication of peer-reviewed reports, to allow the value of nurse-delivered ADHD care to be scientifically verified. This evidence will be invaluable in supporting the growth of nurse-led ADHD services and for those who support greater remuneration for the expanded role of NIPs.
